# Green industry work: production of FeCl_3_ from iron and steel industry waste (mill scale) and its use in wastewater treatment

**DOI:** 10.1007/s11356-024-32451-6

**Published:** 2024-02-17

**Authors:** Alper Solmaz, Ömer Saltuk Bölükbaşi, Zeynel Abidin Sari

**Affiliations:** 1 Department of Environmental Protection and Control-Iskenderun Vocational School of Higher Education, Iskenderun Technical University, Hatay, Turkey; 2 Department of Metallurgy and Materials Engineering, Faculty of Engineering and Natural Sciences, Iskenderun Technical University, 31200 Hatay, Turkey; 3 Department of Metallurgy-Iskenderun Vocational School of Higher Education, Iskenderun Technical University, Hatay, Turkey

**Keywords:** Iron and steel wastewater, Mill scale, Coagulation-flocculation, FeCl_3_ production, Heavy metal

## Abstract

Mill scale (MS) is considered to be a significant metallurgical waste, but there is no economical method yet to utilize its metal content. In this study, which covers various processes in several stages, the solution of iron in MS, which is the Iron and Steel Industry (I&SI) waste, as FeCl_3_ (MS-FeCl_3_) in the thermoreactor in the presence of HCl, was investigated. In the next step, the conditions for using this solution as a coagulant in the treatment of I&SI wastewater were investigated using the jar test. The results of the treated water sample were compared by chemical oxygen demand (COD), total suspended solids (TSS), color, and turbidity analyses using commercial aluminum sulfate (Al_2_(SO_4_)_3_) and FeCl_3_ (C-FeCl_3_). Additionally, heavy metal analyses were conducted, and the treatment performance of three coagulants was presented. Accordingly, while 2.0 mg/L anionic polyelectrolyte was consumed at a dosage of 4.05 mg/L Al_2_(SO_4_)_3_ at pH 7.0, 0.25 mg/L anionic polyelectrolyte was consumed at a dosage of 1.29 mg/L at pH 5.0 in the C-FeCl_3_ and MS-FeCl_3_ studies. Also, Fe, Cr, Mn, Ni, Zn, Cd, Hg, and Pb removal efficiencies were over 93.56% for all three coagulant usage cases. The results showed that the wastewater treatment performance of MS-FeCl_3_ by the recycling of MS, which is an I&SI waste, was at the same level as C-FeCl_3_. Thus, thanks to recycling, waste scale can be used as an alternative to commercial products for green production.

**Graphical abstract:**

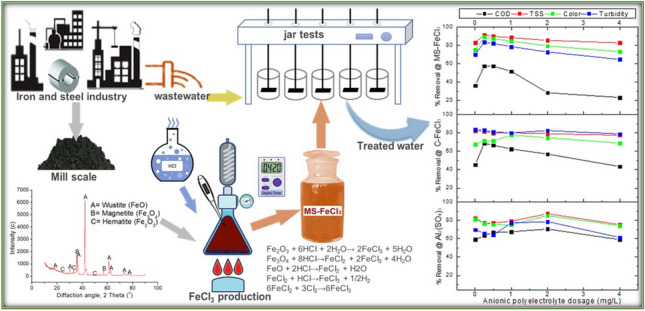

## Introduction

Cooling the billet/plate released to the market in at rolling mill unit within the I&SI is a common method. Meanwhile, oxide layers appear in the high-temperature and oxygen range. This layer is called scale and is classified as waste (Khaerudini et al. 2019). This oxide layer is removed using high-pressure water from the MS formed on the surface due to the high-temperature (1100–1300°C) and oxidizing environment during the production phase (Önkibar 2006; Gündoğdu 2013). Most of the MS is composed of iron oxide structures such as wustite (FeO), magnetite (Fe_3_O_4_), and hematite (Fe_2_O_3_) (Turan et al. 2021). The scale typically contains around 70% Fe (El-Hussiny et al. 2014). Scale is known as an important metallurgical waste, but there is no technology yet to use it economically. On the other hand, in recent years, mostly since the tremendous growth of the I&SI, MS has become a valuable material. MS is now collected and sold for reuse in many industrial applications (Sanin et al. 2019). Although the exact rate is unknown in rolling mills worldwide, 2% of steel production is assumed to involve be scale. If the world’s steel production is 1.950 billion tons, approximately 39 million tons of scale is formed per year (Jikar and Dhokey 2021; World Steel Association 2022). Scale is briquette in integrated iron and steel plants, and it is used with iron ore in basic oxygen furnaces (BOF) and pelletizing plants (Lisin et al. 2003; Baryshev et al. 2011; Osman 2012). By reducing the iron in scale, iron powder (Sen et al. 2015) and Fe_3_O_4_ are produced (Shahid et al. 2019). Additionally, it is possible to encounter the use of scale as a reinforcement material in low-cost ceramic production (Bantsis et al. 2012), electromagnetic wave protectors (Ozturk et al. 2020), and concrete and brick production (Baghel et al. 2020). A part of MS is directly recycled within the steelmaking industry and amounts are used in ferroalloys, cement plants, road construction, to make tiles, to prepare some iron oxide pigments, and in the petrochemicals industry (Iluiu-Varvara et al. 2020). MS contains high amounts of iron. Therefore, it can be used to recover iron by mixing it with industrial waste and other by-products to create the raw material used during the production process. In these applications, MS is processed by roasting, leaching and other processes. As a result, the utilization of the scale formed in I&SI is a common problem of similar industries worldwide. Since the recycling of scale allows the reduction of raw material dependency and the improvement of the ecology, reusing scale is becoming more important due to the constant increase in the cost of iron ore. Therefore, there is a great trend towards recovering valuable iron content in the production process. It has become a subject in recent years that researchers frequently focus on.

I&SI are industrial establishments that use many raw material resources and release high amounts of solid, liquid, and gaseous wastes to the environment in the same proportion. The aforementioned industrial sector wastewaters contain significant amounts of heavy metals originating from various production or cooling processes, and the most common metals: Fe, Pb, Cd, Cu, and Zn (Ahmaruzzaman 2011; Iftikhar et al. 2023; Pang et al. 2023). In the process of pig iron production at high temperatures, there is a large amount of water consumption in the cooling section, steel shaping sections, and coating units due to the nature of the process. Before the actual processing step of steel production, clean water is supplied to the water supply system and then transported to steelmaking plant sections such as coking, sintering, blast furnace, steel mill, hot rolling mill, and cold rolling mills sections. While a small part of the water is consumed in the process in the factory, a large part of it is sent at the wastewater treatment plant, where it is treated and classified according to the treatment method used. If the discharged wastewater can be improved and reused, serious benefits can be achieved in terms of sustainability.

The steel production in the world in 2022 amount to 1.95 billion tons, and Turkey ranks 7th in the world and 1st in Europe with 40.4 million tons of steel produced (SteelData 2023). It is assumed that water reuse, water consumption, and water discharge per ton of steel in iron and steel plants are 14.93 t, 5.90 t, and 9.23 t, respectively (Tong et al. 2019). While the world’s water consumption per ton of steel is between 3 and 6 m^3^, it can reach 25–60 m^3^ in less developed countries (Sirajuddin et al. 2010). Process water per ton of raw steel in Turkey’s I&SI varies between 3.5 and 15 m^3^. In this regard, in the sections of plants where water is consumed in the process, after a few cycles (varying depending on the production amount and raw material input), the water is sent to a wastewater treatment plant.

I&SI cooling water and water originating from other washing processes contain metal particles, suspended solids, oil-grease, organic matter, ammonia, surfactant, cyanide, fluoride, metal, heavy metal, and especially ferrous ions (Biswas 2013; Mahjouri et al. 2017; Phan Quang et al. 2022). The pollutants in these wastewaters are treated by adsorption (Beh et al. 2012), the Fenton process (Phan Quang et al. 2022), the membrane filtration method (Changmai et al. 2020; Zhang et al. 2022b), ultrafiltration and reverse osmosis (Sun et al. 2019), and coagulation-flocculation processes (Garg and Singh 2022). The net surface charges are reduced by adding metal salts to the negatively charged particles repel each other in water that. With sufficient Van der Walls forces, the pollutants are precipitated by reducing the surface area and increasing their weight by allowing them to hold onto each other. This process is known as the coagulation process. It is known that among the treatment processes listed above, scale-up is preferred more than other processes in terms of cost and the ease of application (Abujazar et al. 2022a). Coagulation is performed by adding chemicals such as Al_2_(SO_4_)_3_, FeCl_3_, ferric sulfate, poly aluminum chloride (PACl), and polyaluminum ferric chloride (PAFCl) as positively charged metal salts (Verma et al. 2012). Polyelectrolytes ensure that are formed are tightened, their molecular weights increase, and flocs are formed. These flocs are removed from the medium by filtration, flotation, or sedimentation (Bratby 2016; Abujazar et al. 2022a). Moreover, in the metal industry, this process is used for the removal of organic and inorganic pollutants from oil and petrochemical industry wastewater (Zhao et al. 2021), the removal of heavy metals such as Cu^2+^, Cr^6+^, Cd^2+^, and Pb^2+^ from many different wastewaters (Fu and Wang 2011), color removal from textile wastewater (Verma et al. 2012), and paper recycling wastewater (Mainardis et al. 2022) and the removal of microplastics (Tang et al. 2022).

While wastewater is created in high amounts in terms of the flow rate in I&SI, it has a negative impact on the receiving environment due to the multiple pollutants (organic wastes and heavy metals) it contains (Mondal et al. 2019; Sun et al. 2019; Garg and Singh 2022). The coagulation/flocculation process, which is one of the safest and most frequently used methods for the treatment of these wastewaters, is a preferred operation. This process requires salts/solutions of metals such as Fe and Al (Das et al. 2018). Like almost all industries, the main goal of I&SI is to be sustainable, especially by reducing the amount of solid waste it produces (Mondal et al. 2019). In this context, the conversion of scale, which contains high amounts of Fe, into FeCl_3_ by thermochemistry and its removal from the wastewater into which it is discharged will make a significant contribution to the basic sustainability targets of this industry.

In this respect, it is clear that various salt compounds based on iron and aluminum are used in the treatment of industrial wastewater today. Examples of these include the iron and steel industry (Jung et al. 2006; Colla et al. 2016; Vasilenko and Koltun 2017), the galvanic, tinned and colored steel sheet processing process (Taheriyoun et al. 2020), and the coating sector from metal side branches. It was treated using metal salts and derivatives (Al and Fe) (Al-Shannag et al. 2015; Oden and Sari-Erkan 2018) in the treatment of wastewater. However, the consumption costs of these products used commercially in treating constitute the most important limitation of the current situation in this field. For this reason, in the study, it was revealed that it is necessary to demonstrate the treated of I&SI wastewater in the presence of Fe^3+^ ions obtained by the extraction method from MS, without the need for any industrial metal salt compounds, and to compare the treatment efficiency performances and polyelectrolyte consumptions of their commercially available counterparts. In addition, by pioneering the development of a method that can replace the metal salt reactants that are frequently purchased and used commercially with the traditional refining method, environmentally risky smelter wastes have also been evaluated. Thus, the environmental and economic disadvantages caused by solid wastes have been turned into advantages in the treatment of liquid wastes. In addition, due to the limited reuse of MS, the fact that it will be used by producing it as metallic salt solutions will have benefits that will contribute to the reduction of stocks of this substance. Considering that the MS-FeCl_3_ produced in the process can be used in other research subjects or in the treatment systems of facilities, it will provide a separate added value.

The main purpose of this study is to investigate the usability of MS-FeCl_3_ coagulant obtained by extraction method from MS in the treatment of I&SI wastewater. For this purpose, the scale formed in the process was converted into FeCl_3_ and used as a coagulant in the treatment of the wastewater of this industry. The optimum production conditions were determined in the treatment process with HCl, and the treatment performance of the obtained MS-FeCl_3_ was compared to the performance of Al_2_(SO_4_)_3_ and C-FeCl_3_, which are widely used. Thus, it has been revealed that MS can be evaluated and used as an alternative coagulant in the treatment of the wastewater in the market.

## Material and method

### Analytical methods

#### Chemicals

Aluminum sulfate (Al_2_(SO_4_)_3_.18H_2_O, 98%, Sigma-Aldrich), iron (III) chloride (FeCl_3_.6H_2_O ≥ 98.0%, Merck), anionic polyelectrolyte (poly (2-acrylamido-2-methyl-1-propanesulfonic acid-co-acrylonitrile), ~ 95 wt.%, Sigma-Aldrich), sodium hydroxide (NaOH, ≥ 99.0%, Sigma-Aldrich), sulfuric acid (H_2_SO_4_, ≥ 99.99%, Merck), 5-sulfosalicylic acid dihydrate (C_7_H_6_O_6_S.2H_2_O, ≥ 99.0%, Merck), ethylene diamine tetra acetic acid (EDTA) (C_10_H_14_N_2_Na_2_O_8_.2H_2_O, 95.0%, Sigma-Aldrich), potassium permanganate (KMnO_4_, 99%, Merck), and hydrochloric acid (HCl, 36%, Sigma-Aldrich) were used in the study. The specifications of the chemicals that are used in the experiments are presented in Table 1.

**Table 1:** Specific information on chemicals

Chemicals	Physical condition	Formula	Molecular weight (g/mole)	Prepared stock solution	Role in experiments
Aluminum sulfate	Powder	Al_2_(SO_4_)_3_.18H_2_O	666.42 g/mol	8.1 g/L	Coagulation
Iron (III) chloride	Powder	FeCl_3_.6 H_2_O	270.33 g/mol	10.33 g/L	Coagulation
Anionic polyelectrolyte	Powder	Relative high MW, medium anionic charge density	Average Mw 206	1.0 g/L	Flocculation
Sodium hydroxide	Pellet	NaOH	40.00 g/mol	0.1 M	pH adjustment
Sulfuric acid	Liquid	H_2_SO_4_	98.08 g/mol	0.1 M	pH adjustment
5-Sulfosalicylic acid dihydrate	Powder	C_7_H_6_O_6_S.2H_2_O	254.21 g/mol	0.2 M	Titration
EDTA	Powder	C_10_H_14_N_2_Na_2_O_8_.2H_2_O	372.24 g/mol	0.1 M	Titration
Potassium permanganate	Powder	KMnO_4_	158.03 g/mol	0.02 M	Titration
Hydrochloric acid	Liquid	HCI	36.46 g/mol	-	Production of MS-FeCl_3_

#### Analysis of Fe^3+^ and Fe^2+^ in the coagulant produced from mill scale

The determination of Fe^3+^ in the solution obtained at the end of the first-stage experiments was achieved with titrimetric method using the sulfosalicylic acid indicator. In the titration process, EDTA at 99% purity was used. Finally, amount of the spent EDTA solution was noted, and the conversion ratio of dissolved iron to Fe^3+^ was calculated using Eq. (1) below (Gülensoy 1968).

1 $$CR\text{ (\%) =}\frac{A x Bx 5.584 \left(mg\right) {Fe}^{3+} x 100}{ C x D x E}$$

where CR is the “Conversion Ratio” of Fe^3+^/dissolved Fe, A is the volume of EDTA spent (mL), B is the initial solution volume (mL), C is the volume of indicator used (mL), D is the volume of solution used (mL), and E is the amount of total iron (mg).

The potassium permanganate titration method determines the ferrous iron concentration in the final product. The method is based on the following reaction:

2 $${{\text{MnO}}}_{4}^{+}+{5{\text{Fe}}}_{2}^{+}+{8{\text{H}}}^{+}\to {{\text{Mn}}}_{2}^{+}+{5{\text{Fe}}}_{3}^{+}+{4{\text{H}}}_{2}{\text{O}}$$

The ferrous iron concentration can be expressed as below:

3 $${Fe}^{2+}=\frac{\left(V-Vo\right).C.0.5585}{m}$$

where V (mL) is the volume of potassium permanganate consumed at the endpoint, V_0_ (mL) is the volume of potassium permanganate consumed by distilled water at the endpoint, C is the concentration (M) of the standard potassium permanganate solution, m is the mass (g) of a sample, and 0.5585 is the mass of 0.001 mol iron (Li et al. 2009).

#### Wastewater sample collection and analysis

Raw wastewater samples were taken from the input point of the central wastewater treatment plant of İskenderun Organized Industrial Zone (Hatay/Turkey), where I&SI wastewater, with a flow rate of approximately 7000 m^3^/day, which has a physical and chemical treatment process, is collected. The samples were collected and stored in 20-L plastic PE containers at + 4 ℃. pH measurements were made with the WTW 315i pH meter. The color analysis was carried out using a UV–Vis spectrophotometer (Peak E-1000UV, China) in the experiments at 455 nm with to SM 2120 C according to the equation *y* = 0.00073*x*-0.007 (*R*^2^ 0.9996) (AWWA 2017), the TSS analysis was performed according to the equation *y* = 0.000591*x*-0.00725 (*R*^2^ 0.9976) at 810 nm (Karam et al. 2020), and the turbidity analysis was performed at 541 nm using the equation *y* = 0.000637*x*-0.01092 (*R*^2^ 0.9979) (Dotto et al. 2019). Furthermore, the COD analyses were performed at 600 nm according to the equation *y* = 0.000431*x*-0.03872 (*R*^2^ 0.9997) SM5220-D Closed Reflux, Colorimetric Method (AWWA 2017). ICP-MS (Inductively Coupled Plasma Mass Spectrometry-Agilent, 7500A Model) was used to analyze raw and treated wastewater for the presence of heavy metals (Beauchemin 2008).

### Experimental procedure

The experiments that were conducted in the study could be divided into three groups. In the first stage, the MS was processed, while the MS was leached HCl, and a solution with FeCl_3_ was obtained in the second stage investigated. In the third stage, the usage conditions of the coagulant for the obtained solution were examined in the treatment of the I&SI wastewater. Finally, C-FeCl_3_ and Al_2_(SO_4_)_3_ were obtained to compare their performance to that of the coagulant we produced for use in the third-stage experiments.

#### First stage: pre-processing of MS

**Fig. 1:**
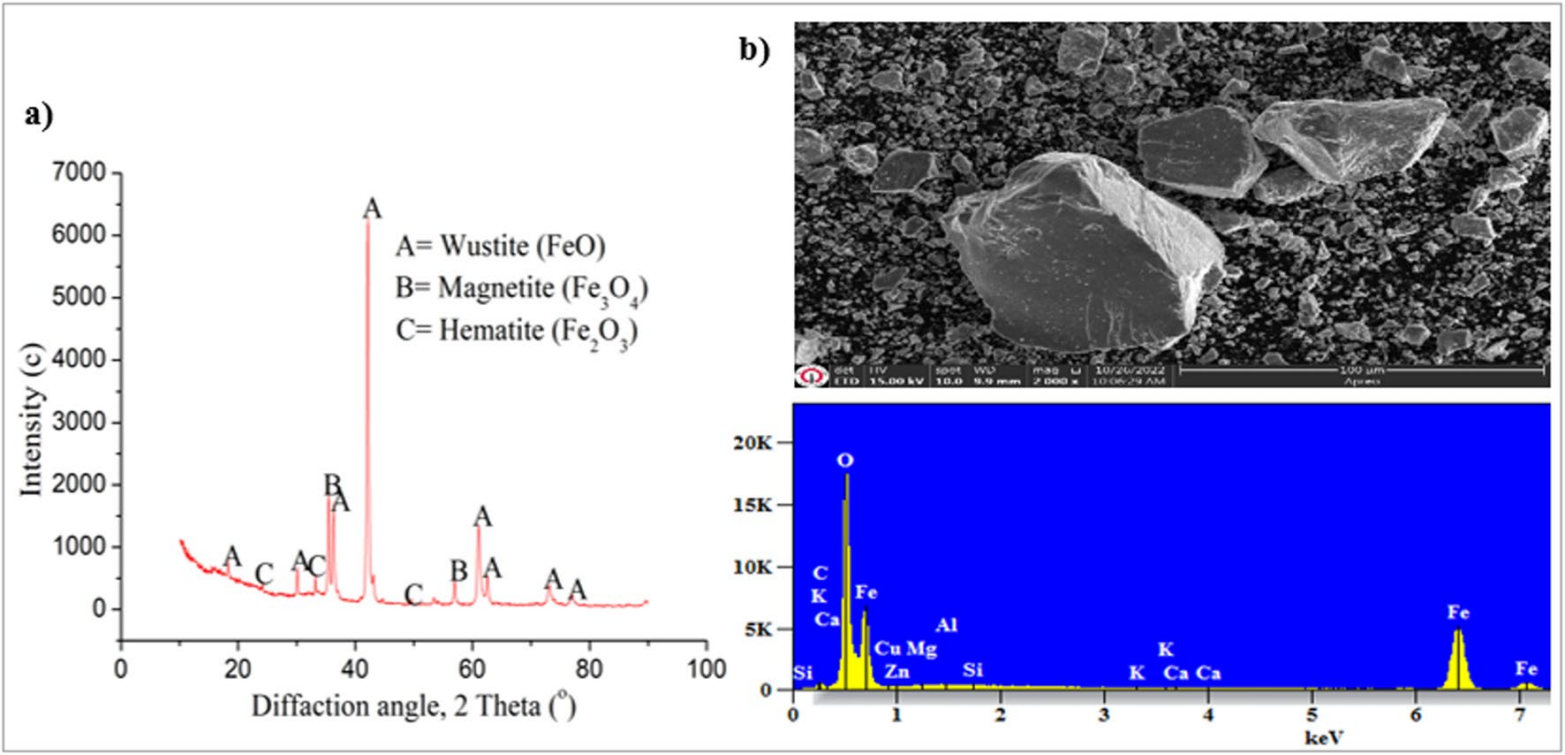
XRD (**a**) and SEM (2000X)-EDX (**b**) images of MS

Approximately 15 kg of MS was obtained from the İskenderun Iron and Steel Factory. Due to the presence of oil, the MS was washed in the laboratory with water a few times and kept at room temperature overnight. Next, after drying for 12 h in a stove adjusted to 323 K and then grinding in a ring mill for 2 min, the sample was passed through a − 90-μm sieve and stored in closed containers to be used later in leaching experiments. The chemical concentrations of the MS were determined by solubilization followed by the analysis of the solutions by ICP-OES (Inductively Coupled Plasma Optical Emission Spectrometry- Perkin-Elmer, Optima 2000DV) (Table 2). It was determined that the chemical composition of the MS was mainly included iron (approximately 64% Fe). The mineralogical analyses of the MS mainly carried out by XRD (X-Ray Diffraction- Bruker/D8 Advance), while their morphological structure was characterized using a scanning electron microscope (SEM—Zeiss/EVO MA10). The XRD and SEM–EDX analysis processes of the MS are seen in Fig. 1. According to the results of the XRD analyses of the MS, the structure mainly consisted of FeO, Fe_2_O_3_, and Fe_3_O_4_. According to the differences obtained in the structural analyses, Fe, which would be the source of the ferric chloride to be obtained from the MS in the presence of HCl, had essentially different oxidation degrees in the MS.

**Table 2:** Chemical analyses of MS

Element	Fe	Al			Cu	Mn	Ca	Ni	Zn	Ga
%	64.020	0.041			0.050	0.480	0.087	0.018	0.018	0.044

#### Second stage: production FeCl_3_ from MS

The first-stage experiments were conducted in a thermoreactor (Hach LT200, Germany) from a heated cylindrical housing. The thermoreactor could be used with 24 borosilicate test tubes with screw cap tubes of at the dimensions 16 × 100 mm. The solutions were prepared by filling a reasonable amount of acid taken from an HCl (37% HCl) stock solution up to 10 mL. At this stage, the effects of various parameters were examined in different ranges for HCl concentration (1–9 M), temperature (318–378 K), and time (15–180 min). After the end of the leaching time, the solution mixture was passed through a blue filter paper. In this study, it was essential to determine the concentration of iron taken into the solution as a result of treating the MS with HCl and on which oxidation step it was. For this reason, in the obtained solutions, Fe^3+^, Fe^2+^, and total Fe analyses were carried out, respectively, by the titrimetric method. Serial experiments were carried out in the determined optimum conditions, and the obtained solutions were stored for use in the third stage of the study.

## Third stage: batch wastewater treatment procedure

Batch jar test experiments were performed at room temperature using a mixer (Velp, Italy) with six beakers containing 50 mL of I&SI wastewater. The experimental procedure included rapid mixing at 240 rpm for 3 min, slow mixing at 60 rpm for 6 min, and settling for 15 min. Al_2_(SO_4_)_3_, C-FeCl_3_, and MS-FeCl_3_ were used as conventional coagulants in the experiments. In the first stage of the coagulation/flocculation study, a 1.0-mg/L flocculant dosage was kept constant, and studies were carried out to determine the optimum pH and optimum coagulant dose for all three coagulants. A total of 0.1 M H_2_SO_4_ and 0.1 M NaOH were used for pH adjustment. In Al_2_(SO_4_)_3_ experiments, 64.8, 32.4, 16.2, 8.1, 4.05, and 0 mg/L were added to each beaker and studied separately at pH 3, 5, 7, 9, and 11. C-FeCl_3_ and MS-FeCl_3_ experiments were also carried out by adding the coagulant at 20.64, 10.32, 5,16, 2.58, 1.29, and 0 mg/L concentrations at the same variable pH values. In all experiments, samples were collected taken from approximately 2 cm below the water surface with the help of a sterile syringe without moving the flocks that had sunk to the bottom. The pH value with the highest efficiency regarding the COD removal parameter of all three coagulants was selected as the optimum, and the second stage was started according to these values. By keeping the optimum pH and coagulant dosages determined at this stage constant, the optimum flocculant dosage was determined based on with variable flocculant dosages (4, 2, 1, 0.5, 0.25, and 0 mg/L).

## Results and discussion

### MS-FeCl_3>_ qualification

#### Effect of HCl concentration on FeCl_3_ production from MS

Two methods are commonly used in the production of FeCl_3_. The first method is the production of FeCl_3_ through the reaction of scrap iron with chlorine gas. The second and more widely used method is the production of FeCl_3_ from dirty acid solutions coming out of the surface cleaning line (pickling line) with HCl during iron-steel production. In general, H_2_SO_4_ is used as an acid in the surface cleaning process. However, recently, the use of HCl has become increasingly widespread. If scale is used as an alternative to ferrous scrap, the production process remains the same as for scrap iron. Additionally, the high iron content of scale and its ability to be ground are advantages in FeCl_3_ production and reaction kinetics. Fe is found in MS at various oxidation steps. Due to its high potential, it is desired that the iron found in the solution as a result of treating the iron in the MS with HCl be mostly in the form of Fe^3+^. Here, it is essential to convert the iron in the MS into various forms of oxidizing ability (Fe^3+^  =  + 0.771 V/Fe^2+^ =  + 0.440 V) in the solution medium and use it in the wastewater of I&SI. The reactions occurring during the formation of FeCl_3_/FeCl_2_ from the MS were as follows:

4 $${{\text{Fe}}}_{2}{{\text{O}}}_{3}+6{\text{HCI}}+{2{\text{H}}}_{2}{\text{O}}\to {2{\text{FeCI}}}_{3}+{5{\text{H}}}_{2}{\text{O}}$$

5 $${{\text{Fe}}}_{3}{{\text{O}}}_{4}+8{\text{HCI}}\to {{\text{FeCI}}}_{2}+{2{\text{FeCI}}}_{3}+{4{\text{H}}}_{2}{\text{O}}$$

6 $${\text{FeO}}+2{\text{HCI}}\to {{\text{FeCI}}}_{2}+{{\text{H}}}_{2}{\text{O}}$$

7 $${{\text{FeCI}}}_{2}+{\text{HCI}}\to {{\text{FeCI}}}_{3}+{1/2{\text{H}}}_{2}$$

Furthermore, in the solution with an oxidizing character to be obtained from MS, in addition to the solubility of iron, the rate of its conversion into Fe^3+^ is also important. Depending on the HCl concentration, the Cl_2_ gas generated during the leaching process would remain in the pressure-resistant sealed glass tubes where the test is performed. This situation will also significantly contribute to the conversion of some FeCl_2_ into FeCl_3_ as described in Eq. (8). In this context, FeCl_3_/FeCl_2_ production from MS in the presence of HCl was investigated under the conditions of various leaching parameters.

8 $${6{\text{FeCI}}}_{2}+{3{\text{CI}}}_{2}\to {6{\text{FeCI}}}_{3}$$

**Fig. 2:**
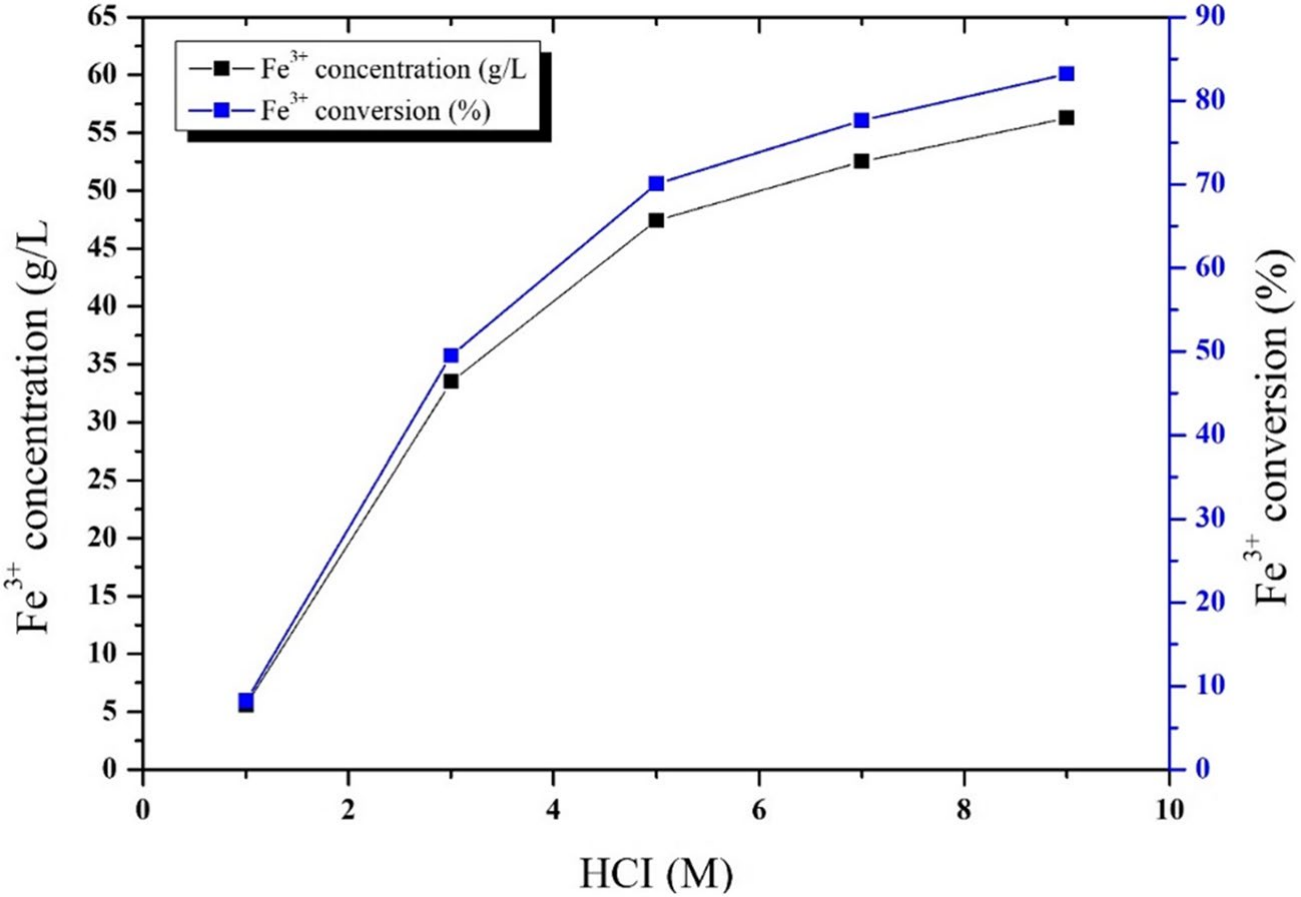
Effect of HCI concentration on the concentration and conversion efficiency of Fe^3+^ from MS (leaching time, 120 min; leaching temperature, 378 K; solid/liquid ratio, 10 (g/mL))

The effect of acid concentration was investigated at 1–9 M HCl concentrations at a constant leaching temperature for 378 K 120 min. Figure 2 shows the effect of acid concentration on Fe^3+^ conversion in the dissolution of iron from MS. The rate of Fe^3+^ concentration was increased by increasing the acid concentration. Fe^3+^ conversion in the solution was initially low but it increased at high concentrations. In the leaching conditions with a leaching temperature of 378 K, a solid–liquid ratio of 1/10 (g/mL), leaching times of 120 min, and HCl concentration of 7 M, it was seen that the ratio of iron taken into the solution was 100%. At the same time, the Fe^3+^ conversion rate was approximately 77%. The results of previous studies (Schwertmann and Taylor 2018) showed that iron oxide dissolution was maximized with HCl solutions between 6 and 12 mol/L. The higher dissolution in HCl was due to the oxidation power of chloride ions. Additionally, the acid concentration is essential for economic concerns.

#### Effect of reaction temperature and time on FeCl_3_ production from MS

**Fig. 3:**
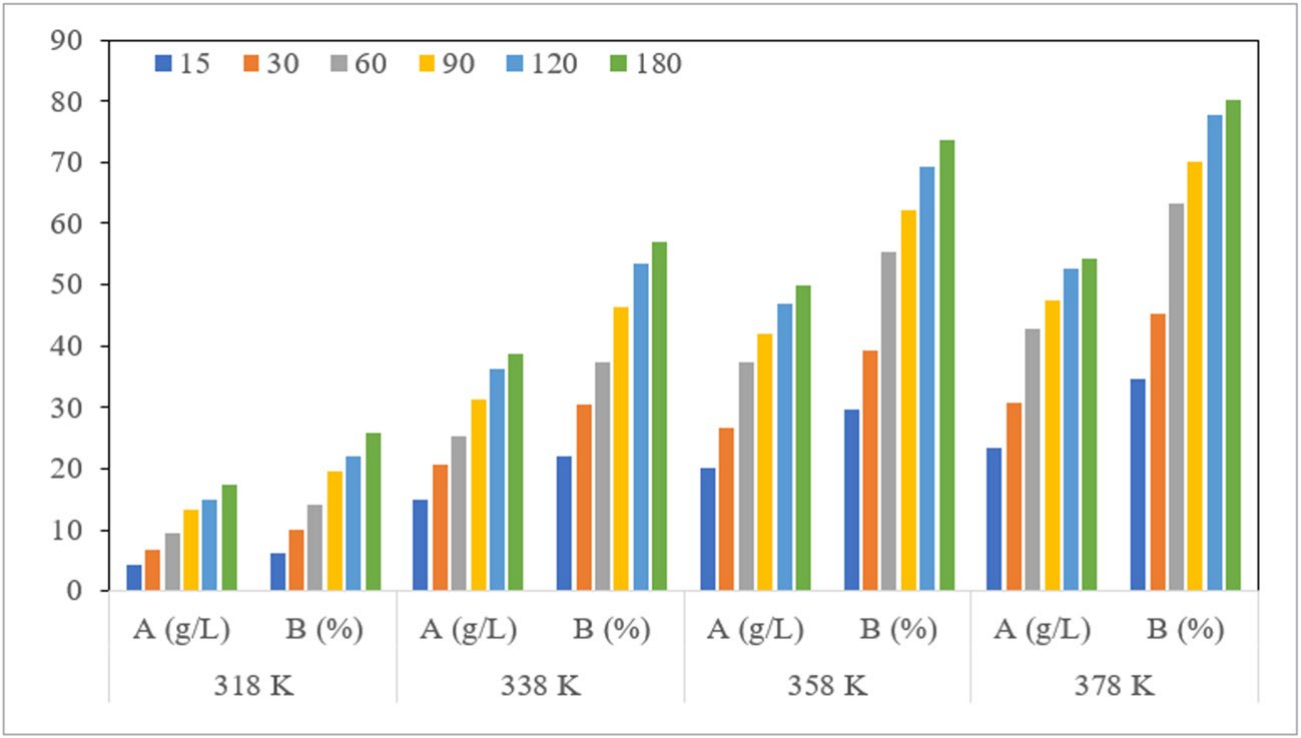
Effect of reaction temperature and time on the concentration and conversion efficiency of Fe^3+^ from MS (HCI concentration, 7M; solid/liquid ratio, 10 (g/mL)); A Fe^3+^ concentration, B Fe^3+^ conversion

Figure 3 shows the effect of contact time on Fe^3+^ conversion as a function of temperature. The Fe^3+^ concentration was increased rapidly with the increase leaching temperature and time. The experiment that was carried out at a temperature of 318 K, for 180 min, at a 7 M HCl concentration and a 1/10 (g/mL) solid–liquid ratio, achieved a 25.9% Fe^3+^ conversion rate. Likewise, at a temperature of 378 K, all iron was taken into the solution, and a Fe^3+^ conversion rate an approximately 80% was observed. Here, the increasing dissolution rate was due to the continued oxidation of metal atoms into metal ions until saturation. Because the iron passing into the solution during the treatment of MS with HCl would be in the forms of both Fe^3+^ and Fe^3+^, it was aimed to identify the conditions with the highest Fe^3+^ conversion rate. Based on this issue, the optimum conditions were determined as a 378 K leaching temperature, a reaction time of 120 min, a solid–liquid ratio of 1/10 g/mL, an HCl concentration of 7 M, and a particle size of − 90 μm. The rate of dissolved Fe was 100% in these conditions, while the Fe^3+^conversion rate and concentration were approximately 77% and 52.53 g/L, respectively. The solutions obtained in these conditions were stored to treat the I&SI wastewater.

**Fig. 4:**
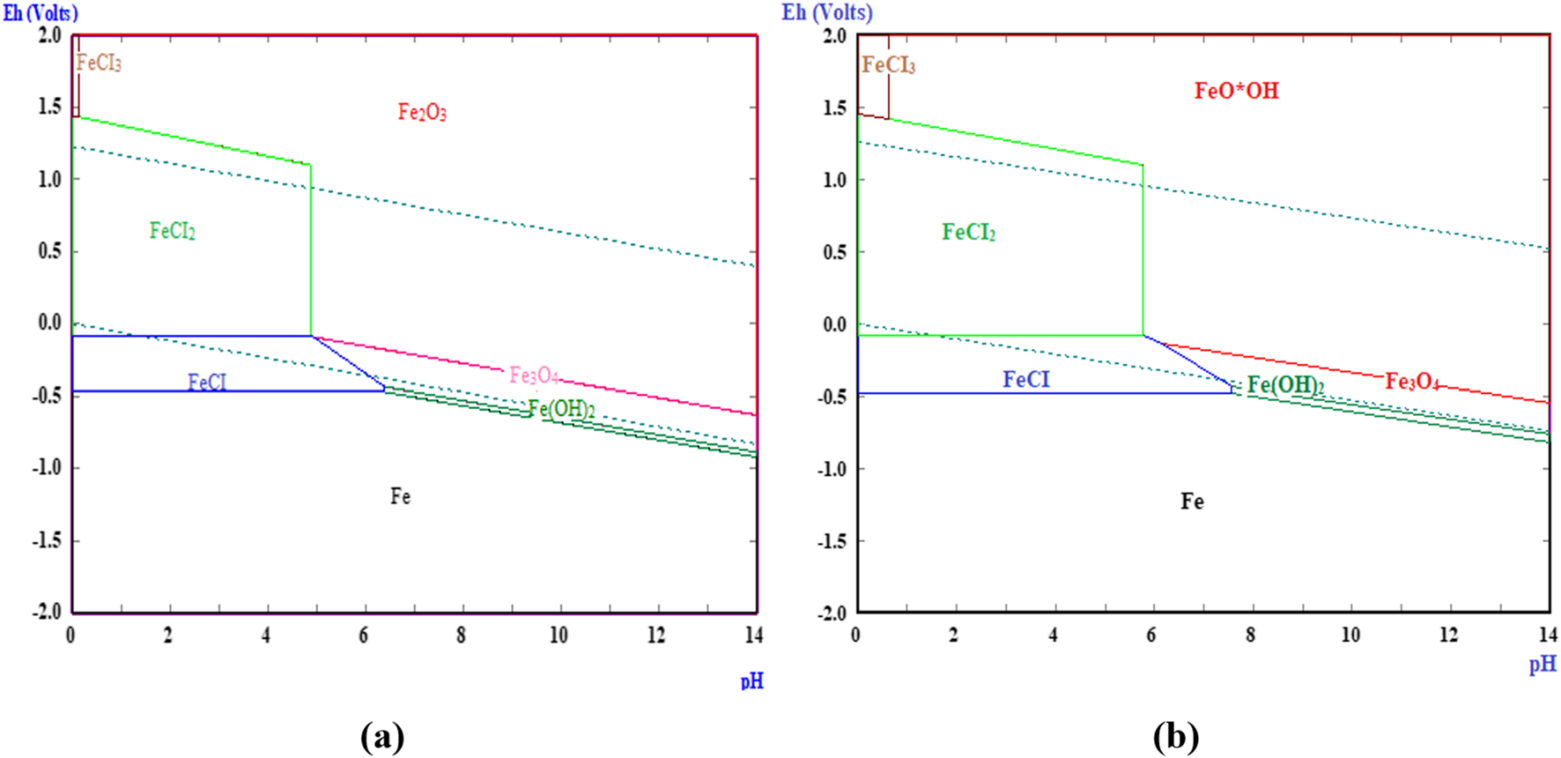
Eh–pH diagram for the Fe-HCI-H_2_O system at (**a**) 318 K and (**b**) 378 K

Moreover, to understand the dissolution behavior of iron from MS under thermoreactor conditions and different temperatures with representative parameters, the Eh–pH diagrams of the possible compounds belonging to the Fe-HCl-H_2_O system were created (Fig. 4). Eh–pH diagrams for the Fe-HCl-H_2_O system were drawn using the HSC/EpH—Eh–pH diagram module. Eh–pH diagrams also offer a quick way of studying the dissolution behavior of different materials. Using the thermodynamic data of the reactions that multiple metals could show in the aqueous medium, the Eh–pH diagram was formed by combining the dissolvability values of oxides and hydroxides and the equilibrium constants of the reactions (Turan et al. 2022). Accordingly, it was understood that FeCl_3_/FeCl_2_ ion species could only be obtained with a decrease in pH and an increase in Eh in the reaction of MS with HCl under the current conditions. However, by the increase in the temperature of the medium, the presence of FeCl_3_ shifted to a broad region. FeCl_3_ was replaced ferric oxyhydroxide (FeO*OH) at high Eh values and all pH values.

#### Characterization of MS leaching solution and leaching residue

At the optimum leaching conditions (leaching temperature of 378 K, leaching time of 120 min, a solid–liquid ratio of 1/10 g/mL, HCl concentration of 7 M), ICP, Raman spectroscopy, and zeta potential measurements to determine the isoelectric point, respectively, in the MS leaching solution were carried out. The results of the ICP analysis of the MS leaching solution obtained under the optimum conditions are shown in Table 3. As seen in Table 3, all elements, except for iron, which was found the scale and completely dissolved, dissolved at specific rates. However, it was determined that their dissolution rates were far below the chemical composition values of facilities that produce FeCl_3_ at the national and international scales.

**Table 3:** Chemical analyses of leaching solution

	Unit	As	Al	Cu	Mn	Cr	Ni	Zn	Sb
This study	(ppm)	0.09	0.86	7.54	0.34	0.04	< 0.05	5.15	0.09
(Koruma Group Companies 2019)	mg/kg %Fe III	Max 20^1^	NA	NA	Max %1^1^	Max 50^1^	Max 60^1^	NA	Max 100^1^
(Altair Chimica 2018)	mg/kg	2.7	NA	NA	Max 0.137	Max 48	Max 48	NA	Max 2.7

*NA* analysis not available

**Fig. 5:**
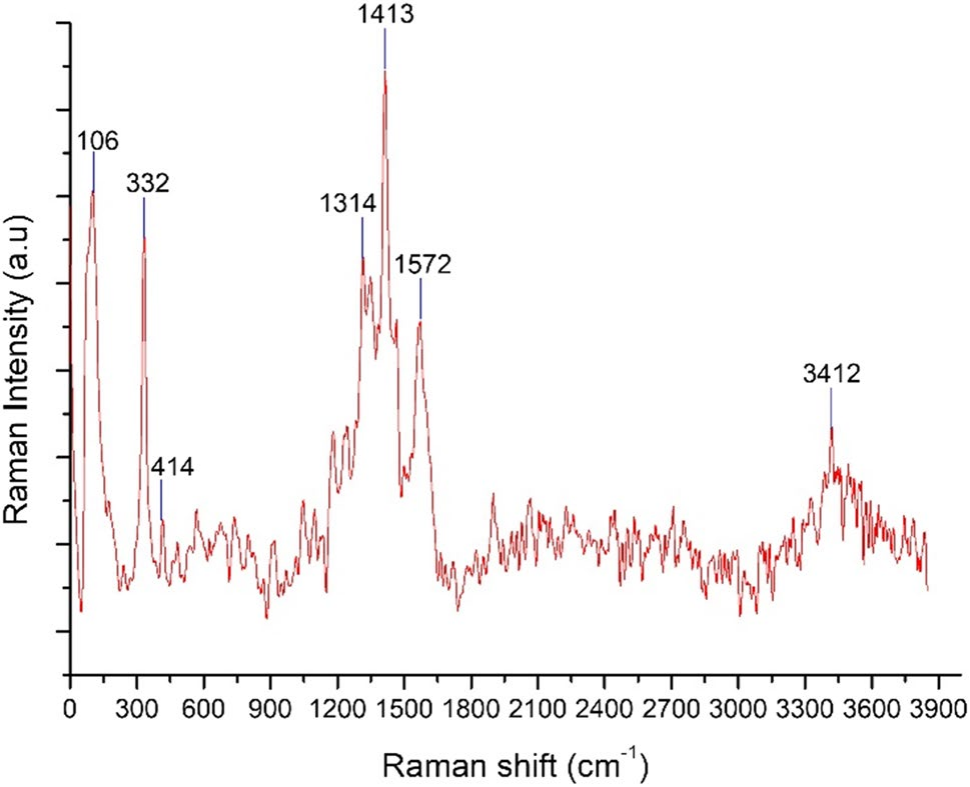
Raman spectra of MS leaching solution (HCI concentration, 7 M; leaching time, 120 min; leaching temperature, 378 K; solid/liquid ratio, 10 g/mL)

The MS aqueous solution samples at the optimum conditions were analyzed by Raman microspectroscopy (WITEc—Alpha M +). Analyses were also carried out with a 532 nm (argon) laser. The Raman signal was collected with a spectral resolution of 1 cm^−1^ over 100–4000 cm^−1^ using × 20 or × 50 microscope lenses. Raman spectroscopy is a non-destructive chemical analysis technique that provides detailed information about chemical structure, phase and polymorphism, crystallinity, and molecular interactions. It is based on the interaction of light with the chemical bonds within a material. Raman spectroscopy is a sensitive method to characterize different Fe^2+^ and Fe^3+^ species structures in aqueous solutions. The results of the Raman analyses of the leaching solution obtained under the optimum leaching conditions indicating different phases are shown in Fig. 5. The Raman analysis showed the coordination of Fe^3+^ and Fe^2+^ with Cl^**−**^**.**

The analysis of the Raman spectra indicated that the peak at ~ 332 cm^−1^ corresponded to FeCl_3_ and FeCl_4_ ([Fe(H_2_O)_6_]^3+^  + 4Cl^−^ = [FeCl_4_]^−^ + 6H_2_O); the peak at 414 cm^−1^ corresponded to Fe_2_Cl_6_; the peak at 1413 cm^−1^ corresponded to Fe^3+^-OH_2_; the peaks at 1314, 1572, and 3412 cm^−1^ corresponded FeCl_2_; and the peak at 2800–3800 cm^−1^ corresponded to H_2_O/OH vibrational mode (Givan and Loewenschuss 1977; Voyiatzis et al. 1999; Baumgartner 2009; Kong et al. 2011; Takaya et al. 2019; Zeng et al. 2020). The two peaks observed below 200 cm^−1^ were the characteristic Raman bands common to all halide ion solutions, which arise from the translational vibrations of water molecules that are hydrogen-bonded to either halide ions or other water molecules (Kanno and Hiraishi 1982).

Zeta potential measurements were made with a Zetasizer-Malvern (Nano ZS90) device at room temperature to determine the isoelectric point of the MS aqueous solution sample under the optimum conditions. The pH at the point where the zeta potential is zero is defined as the isoelectric point (pip). The isoelectric point is where the system is the most unstable. In the analyses conducted accordingly, it was determined that the isoelectric point was in the acidic region (pH:3.1). The zeta potential also became more negative as the pH of the solution increased.

### Batch wastewater treatment results

#### Determination of optimum pH, coagulant dosages, and flocculant dosages

pH is a significant factor in the coagulation process. Each coagulant has a pH value at which it is active. A small change in pH can stabilize or destabilize dispersions. Pollutants in dissolved or colloidal form in water are subject to chemical surface changes in every pH range (López-Maldonado et al. 2014). When a metal is added to water at a certain pH, dissolution occurs. Metal (M) ions present in water are hydrolyzed to form monomeric, polymeric, and solid precipitates as follows: M(OH)_2_^+^, MOH^2+^, M_2_(OH)_2_^4+^, M(OH)_4_^5+^, M(OH)_3_^0^(s), and M(OH)_4_^−^ (Dentel and Gossett 1988; Verma et al. 2010). In particular when Fe salts are added, the pH value decreases. The metal precipitates from the optimum pH range of 3–6 (Eq. 9) (Verma et al. 2010). Therefore, pH needs to be adjusted for the optimum efficiency (Amuda and Amoo 2007; Qiao et al. 2012).

9 $${{\text{FeCl}}}_{3}+{3{\text{H}}}_{2}{\text{O}}\to {{\text{Fe}}\left({\text{OH}}\right)}_{3\left({\text{s}}\right)}\downarrow +{3{\text{H}}}^{+}+{3{\text{Cl}}}^{-}$$

Additionally, a decrease in pH appears with the addition of aluminum salts, which reduces the efficiency of precipitation. The optimum precipitation efficiency was observed in the pH range of 5.7–7 (Eq. 10) (Benjamin 2014).

10 $${{\text{Al}}}_{2}{\left({{\text{SO}}}_{4}\right)}_{3.}{14{\text{H}}}_{2}{\text{O}}\to {2{\text{Al}}\left({\text{OH}}\right)}_{3\left({\text{s}}\right)}\downarrow +{{3{\text{SO}}}_{4}}^{-2}+{6{\text{H}}}^{+}+{8{\text{H}}}_{2}{\text{O}}$$

**Fig. 6:**
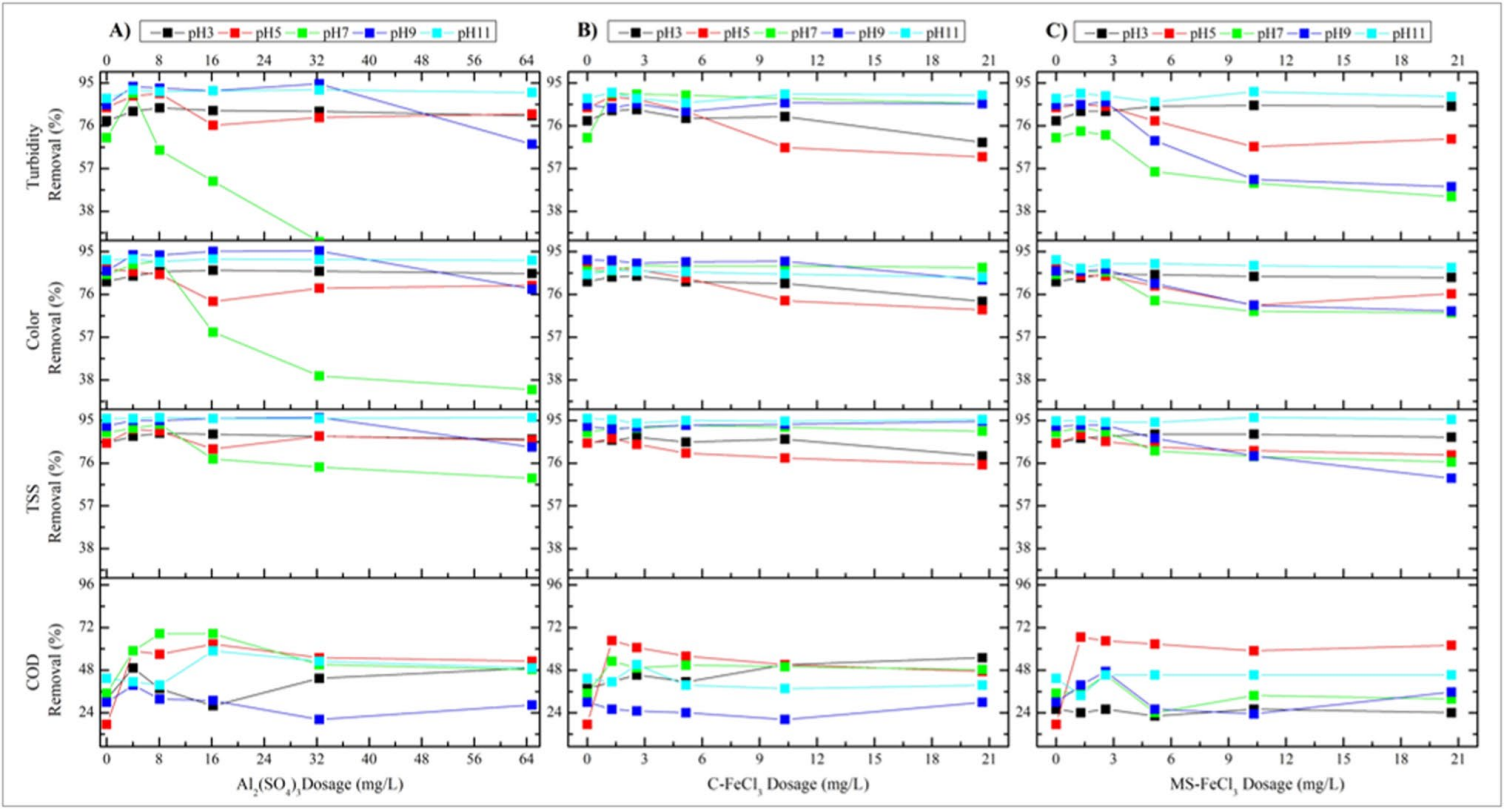
COD, TSS, color, and turbidity removal rates at different pHs, varying coagulant dosages, and a fixed 1.0 mg/L anionic polyelectrolyte dosage. **A** Al_2_(SO_4_)_3_. **B** C-FeCl_3_. **C** MS-FeCl_3_

To determine the optimum pH effect in the performance trials of the Al_2_(SO_4_)_3_, C-FeCl_3_, and MS-FeCl_3_ coagulants, a fixed anionic polyelectrolyte dosage (1.0 mg/L) was first studied. Long-chain polymeric flocculants attract particles formed with metal salts by adsorption, and they are used to ensure the faster precipitation of these particles by increasing their size and weight (Wei et al. 2018). As the effect of precipitation time on precipitation efficiency was negligible as a result of the preliminary experiments, the precipitation time variable was not studied (Dotto et al. 2019). The COD, TSS, color, and turbidity removal efficiencies at variable pH values in the removal studies using the aforementioned coagulants are presented in Fig. 6.

**Fig. 7:**
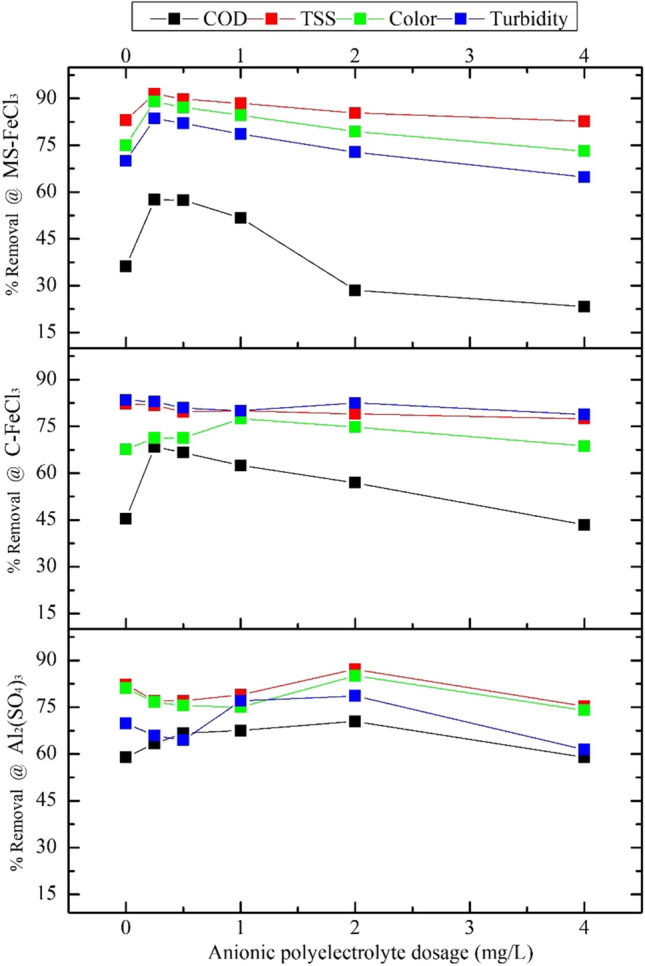
Study results to determine the optimum anionic poly dosage

In the process outlined in Fig. 6-A, the dosage of Al_2_(SO_4_)_3_ as the coagulant was kept in the range of 0–16 mg/L, and 1.0 mg/L anionic polyelectrolyte was added as a flocculant. As seen in Fig. 7, in the removal study with Al_2_(SO_4_)_3_, the most effective pH in COD removal was 7.0, although there was still removal of turbidity, color, and TSS, except for pH 7.0, below and above the neutral among the different pH values. On the other hand, as the dosage of Al_2_(SO_4_)_3_ increased, it is clearly seen that the yield did not increase in every parameter, but decreased. When the Al_2_(SO_4_)_3_ dosage was increased from 0 to 4.05 mg/L at pH 7.0, the removal efficiency of COD was around 50%, but when the dosage was 8.1 mg/L, the efficiency rose to around 70%. Nevertheless, it can be easily seen that the efficiency value did not increase when the Al_2_(SO_4_)_3_ dosage was increased. Therefore, it was unnecessary to dose more Al_2_(SO_4_)_3_. In summary in the presence of 4 mg/L Al_2_(SO_4_)_3_, turbidity, color, and TSS removal increased above 80% at almost every pH value, while COD removal efficiency was calculated as 68.52% in the presence of 4 mg/L Al_2_(SO_4_)_3_ at pH 7.0. Using dosages in the range of, after 0–22 mg/L C-FeCl_3_ at variable pH values (Fig. 6-B), 1.0 mg/L anionic polyelectrolyte was added again. In the removal study with C-FeCl_3_, turbidity, color, and TSS removal was observed, except for pH 5.0, but it can be seen that this was the most effective pH value for COD removal. It can be seen that the removal efficiency of COD reached around 70% when the C-FeCl_3_ dosage is increased from 0 to 1.29 mg/L at this pH, but the efficiency decreased when the dosage was increased further. Therefore, it is clear that further dosing of C-FeCl_3_ was also unnecessary. Accordingly, at low C-FeCl_3_ dosages (below 2 mg/L), the removal efficiencies of turbidity and color were high than 70%, while the yield tended to decrease when the dosage was increased. On the other hand, it was clear that the removal efficiencies were high at pH 11. However, this is due to the precipitation of calcium ions naturally found in water as CaCO_3_ at high pH, as well as the precipitation of magnesium ions as Mg(OH)_2_ (Semerjian and Ayoub 2003). Therefore, the COD removal efficiency was calculated as 64.66% in the presence of 0.0125 mg/L C-FeCl_3_. In the coagulation experiments with MS-FeCl_3_ produced from the MS examined, the exact dosages of C-FeCl_3_ were applied. The plot curves were found to be very similar to the curves in the C-FeCl_3_ studies, as seen in Fig. 6. In the removal study with MS-FeCl_3_, as in the C-FeCl_3_ dosage studies, removal was observed in 3 parameters except pH 5.0. Considering the COD parameter, on the other hand, it can be seen that the most effective pH was again 5.0. Again, it was seen that the removal efficiency of COD reached around 70% when the MS-FeCl_3_ dosage increased from 0 to 1.29 mg/L at this pH. However, it was clearly seen that the efficiency decreased when the dosage was increased further. Therefore, further dosing was unnecessary in the MS-FeCl_3_ study as it was in the C-FeCl_3_ study. In summary, the COD removal efficiency was calculated as 66.59% in the presence of 0–22 mg/L MS-FeCl_3_ (in Fig. 6-C). Moreover, the optimum conditions for the highest COD removal when Al_2_(SO_4_)_3_ was used as the coagulant was 4.05 mg/L at pH 7.00 among the varied pH values. The optimum conditions for the use of both the C-FeCl_3_ and MS-FeCl_3_ coagulants were a dose of 1.29 mg/L and a pH value of 5.00. The optimum pH and dosage value amounts were found by evaluating the removal efficiencies of all three coagulants based on COD. By keeping these values constant, optimum anionic polyelectrolyte content experiments were carried out.

In Fig. 7, it is seen that the anionic polyelectrolyte dosage was kept in the range of 0–4 mg/L at 4.05 mg/L Al_2_(SO_4_)_3_ and at pH 7.0. As the dosage increased up to 2.0 mg/L, the yield also increased, but the yield decreased when the dosage was raised further. The COD, TSS, color, and turbidity removal efficiencies at 2.0 mg/L of polyelectrolyte were calculated as 70.46, 87.13, 85.06, and 78.57%, respectively. It was observed that the efficiency decreased when the anionic polyelectrolyte dosage was increased to the dosage of 1.29 mg/L C-FeCl_3_ at pH 5.0, and the best efficiency was obtained at the dosage of 0.25 mg/L polyelectrolyte. In these dosages, the COD, TSS, color, and turbidity removal efficiencies were calculated as 68.52, 81.85, 71.22, and 82.96%, respectively. Additionally, in the MS-FeCl_3_ study, a situation similar to the C-FeCl_3_ study emerged, and the COD, TSS, color, and turbidity removal efficiencies in the presence of 1.29 mg/L MS -FeCl_3_ and 0.25 mg/L polyelectrolyte dosage were 57.53, 91.53, 89.05, and 83.55, respectively. In the removal mechanism examined, trivalent aluminum and iron cations, which are metal salts entering the aqueous medium, had very similar characteristics. When these salts are dissolved in water, hydrates of metal (M) ions are formed and hydrolyzed to monomeric or polymeric species (MOH^+2^, M(OH)^2+^, M(OH)_2_^4+^, M(OH)_4_^+5^, M(OH)_3_, and M(OH)_4_^−^). Under acidic conditions, these salts are in the solution, but when the pH is increased or the concentrations of these salts increased, ferric hydroxide (Fe(OH)_3(s)_) or aluminum hydroxide (AI(OH)_3(s)_) forms are created. In other words, all metals have the minimum solubility if there are enough coagulants in the environment and at a pH where there is minimal solubility. Likewise, these create hydroxide forms with a gelatinous appearance. These forms are amorphous, they are hydrophobic, have a large surface area, and are positively charged. Since pollutants in the water generally have a negative charge, they are adsorbed on the surfaces of these formed metal particles and become insoluble. Immediately after this process, when precipitation occurs, pollutants are removed (Stephenson and Duff 1996; Pang et al. 2009; Bratby 2016). As a result of evaluating all three coagulants based on their optimum COD removal efficiency values, the optimum pH and dosage values were determined, and optimum anionic polyelectrolyte were found by carrying out experiments while keeping these values constant.

#### Heavy metal removal efficiencies and toxicological assessment under optimum conditions

**Fig. 8:**
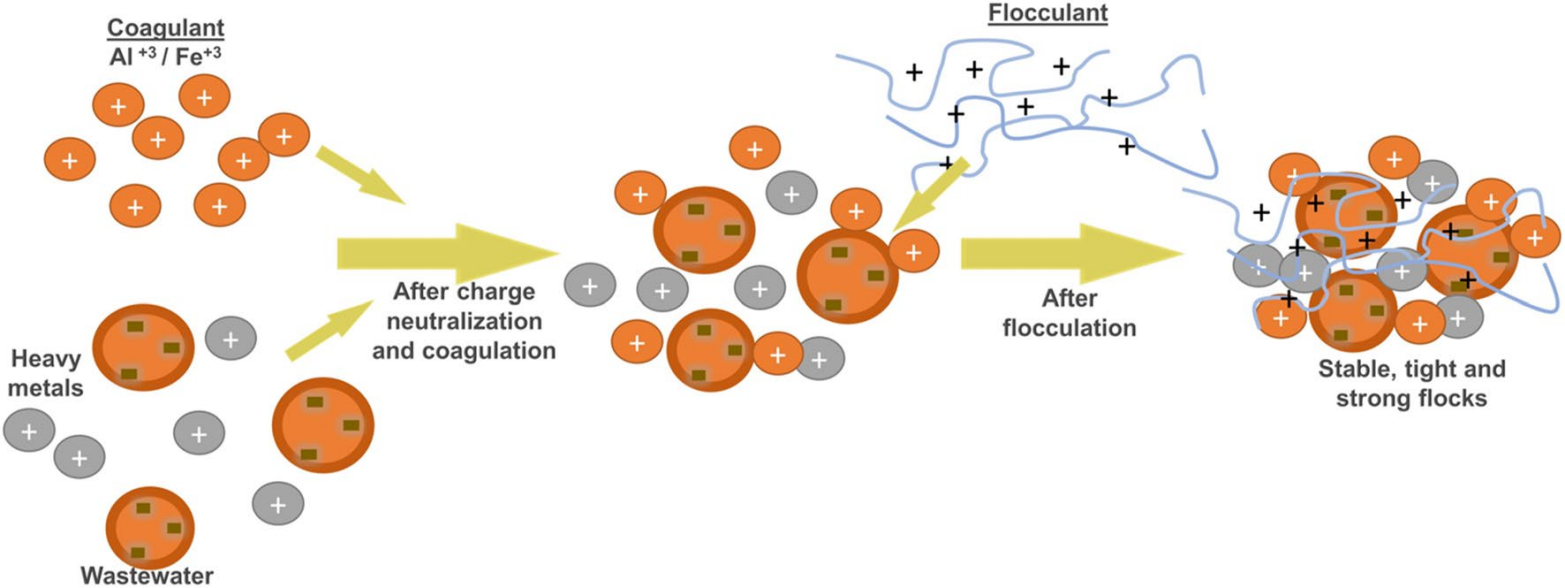
Visual of coagulation/flocculation mechanism

The results of the analyses of the effluents obtained at the end of the jar tests with the three coagulants and the results of the analyses of the raw wastewater are presented in Table 4. In the raw wastewater, Zn, Fe, Mn, and Pb concentrations were high. As a result of the treatment studies with Al_2_(SO_4_)_3_, more than 96.6% efficiency was obtained in the removal of other metals. The treatment results with C-FeCl_3_ showed a removal efficiency of over 94.6%. The treatment studies with MS-FeCl_3_ achieved a treatment efficiency of over 94.5%. Again, the heavy metal removal mechanism acted as a general precipitation mechanism. The visual representation of this mechanism is presented in Fig. 8. That is, at the optimum pH where Fe(OH)_3(s)_ and Al(OH)_3(s)_ ions, which become polymerized and have a high surface area, do not dissolve, heavy metals are adsorbed onto these hydroxides and precipitate out together (Pang et al. 2009; Dubery et al. 2017).

**Table 4:** Heavy metal removal results

Parameter	Unit	Raw wastewater	Treated wastewater		
			Al_2_(SO_4_)_3_	C-FeCl_3_	MS-FeCl_3_
pH	-	7.9	-	-	-
COD	mg/L	120	35.45	37.77	48,65
TSS	mg/L	384.55	87.13	69.80	32.58
Color	Pt–Co	654	97.70	188.25	71.63
Turbidity	mg/L	409.55	87.77	69.80	67.37
Fe	µg/L	20,000	2.12	94.18	41.07
Cr	µg/L	100.01	3.04	1.07	0.05
Mn	µg/L	1410	17.72	90.86	21.65
Ni	µg/L	30.00	0.16	0.82	1.65
Zn	µg/L	33,890	370	1830	969
Cd	µg/L	210.02	1.06	7.56	5.45
Hg	µg/L	1.01	0.02	0.01	0.01
Pb	µg/L	5,840	1.23	19.34	0.57

In the examinations regarding Zn, which is one of the primary metal pollutants, the removal efficiency was found to be 98.91% at the dosage of 4.05 mg/L Al_2_(SO_4_)_3_ and 2.0 mg/L anionic polyelectrolyte. Following the same amount (1.29 mg/L) of C-FeCl_3_ and the same MS-FeCl_3_ dosage, the removal efficiencies of Zn were calculated as 94.60% and 97.14%, respectively, at the 2.0 mg/L anionic polyelectrolyte dosage. Furthermore, the removal efficiency of Fe was over 99.99% in the case of the addition of Al_2_(SO_4_)_3_ at the aforementioned doses. In comparison, 99.53% and 99.79% efficiency values were obtained by the same dosages of C-FeCl_3_ and MS-FeCl_3_, respectively. Additionally, the highest Pb removal efficiency was seen in the case of using MS-FeCl_3_ as 99.99%. Again, the removal efficiency of Mn using MS-FeCl_3_ was calculated as 98.46%, while the removal efficiencies obtained when Al_2_(SO_4_)_3_ and C-FeCl_3_ were used were 98.74% and 93.56%, respectively. If a general evaluation is made in terms of heavy metal removal efficiencies, these efficiencies were very close to each other at the same value (2.0 mg/L) of anionic polyelectrolyte dosage, with the same dosages (1.29 mg/L) of C-FeCl_3_ and MS-FeCl_3_.

As it is known, heavy metals have high atomic weight. They also have a specific gravity at least 5 times greater than the specific gravity of water. Toxic metals, including heavy metals, are metal compounds that have a negative impact on human and environmental health. The toxicities of these metals depend on dosage, type of meat, and exposure. It also depends on the specific characteristics of the exposed creature (age, gender, genetic factors, and feeding habits). Most known metals, especially heavy metals, are toxic, but some are essential and some, such as bismuth, are slightly less toxic than others (Tchounwou et al. 2012). On the other hand, metals with abnormal oxidation can also become toxic: For example, chromium (III) is an essential trace element, but chromium (VI) is known to be carcinogenic. Moreover, compounds containing soluble metals show toxic properties. Soluble metals are called coordination complexes consisting of a metal ion surrounded by ligands (Ali et al. 2013).

Heavy metals act toxically after a certain concentration (1–10 ppm) (Fe, Cu, Zn, Ni, and Se) (Järup 2003). On the other hand, this concentration value varies depending on the presence of the heavy metal in soil and water. These limit values for some metals in soil are given by the World Health Organization (WHO): Ni 80, Cu 30, Cr 100, Mn 200, As 20, Sb 36, Fe 50,000, and Zn 300 ppm (WHO 1996). In this study, as seen in Table 3, the ratio of some metals found in the leach solution and included in the heavy metal class is given. It has been determined that at the end of the treatment, some or all of these metals precipitate and remain in the sewage sludge, and these metals do not have any toxic effects compared to the rates that can be found in the soil, according to the WHO. On the other hand, according to the WHO, the maximum acceptable concentrations of some toxic metals in drinking water for copper, nickel, arsenic, antimony, manganese, zinc, aluminum, mercury, lead, iron, and chromium are given as 2, 0.07, 0.01, 0.02, 0.05, 3, 0.2, 0.006, 0.01, 3, and 0.05 mg/L respectively (WHO 2022). As a result of the jar test performed with MS-FeCl_3_ in this study, it shows that the toxic metal rates in the treated wastewater are below acceptable values, as seen in Table 4. This proves that treated wastewater has no toxicity effect according to the WHO.

#### Sludge characterization

**Fig. 9:**
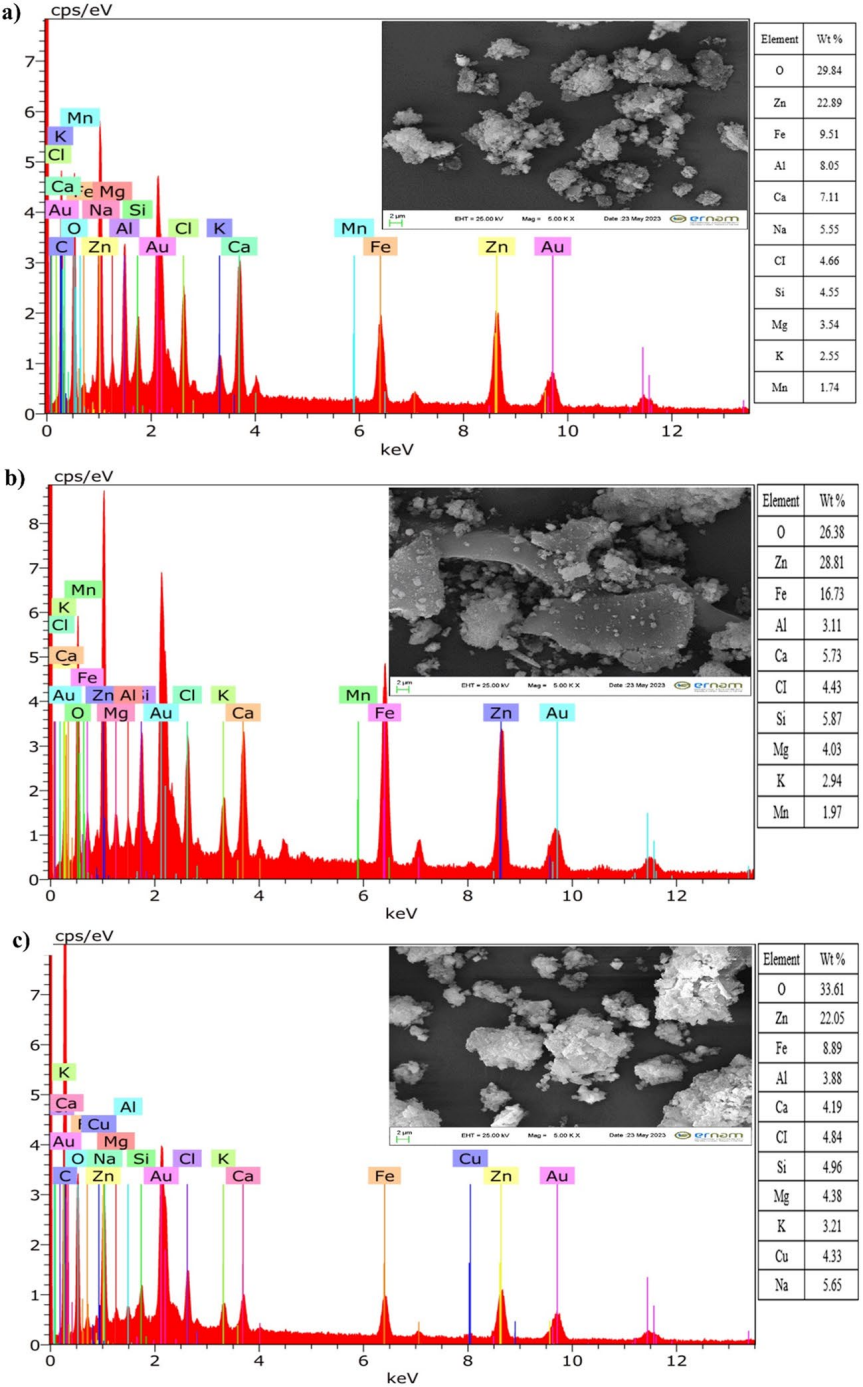
SEM (× 5000) micrographs and EDX spectrums of sludge formed during coagulation (**a**) Al_2_(SO_4_)_3_, (**b**) C-FeCl_3_, and (**c**) MS-FeCl_3_ (optimum condition for Al_2_(SO_4_)_3_ of 4.05 mg/L and anionic polyelectrolyte of 2.0 mg/L, for C-FeCl_3_ and MS-FeCl_3_ of 1.29 mg/L and anionic polyelectrolyte of 0.25 mg/L)

**Fig. 10:**
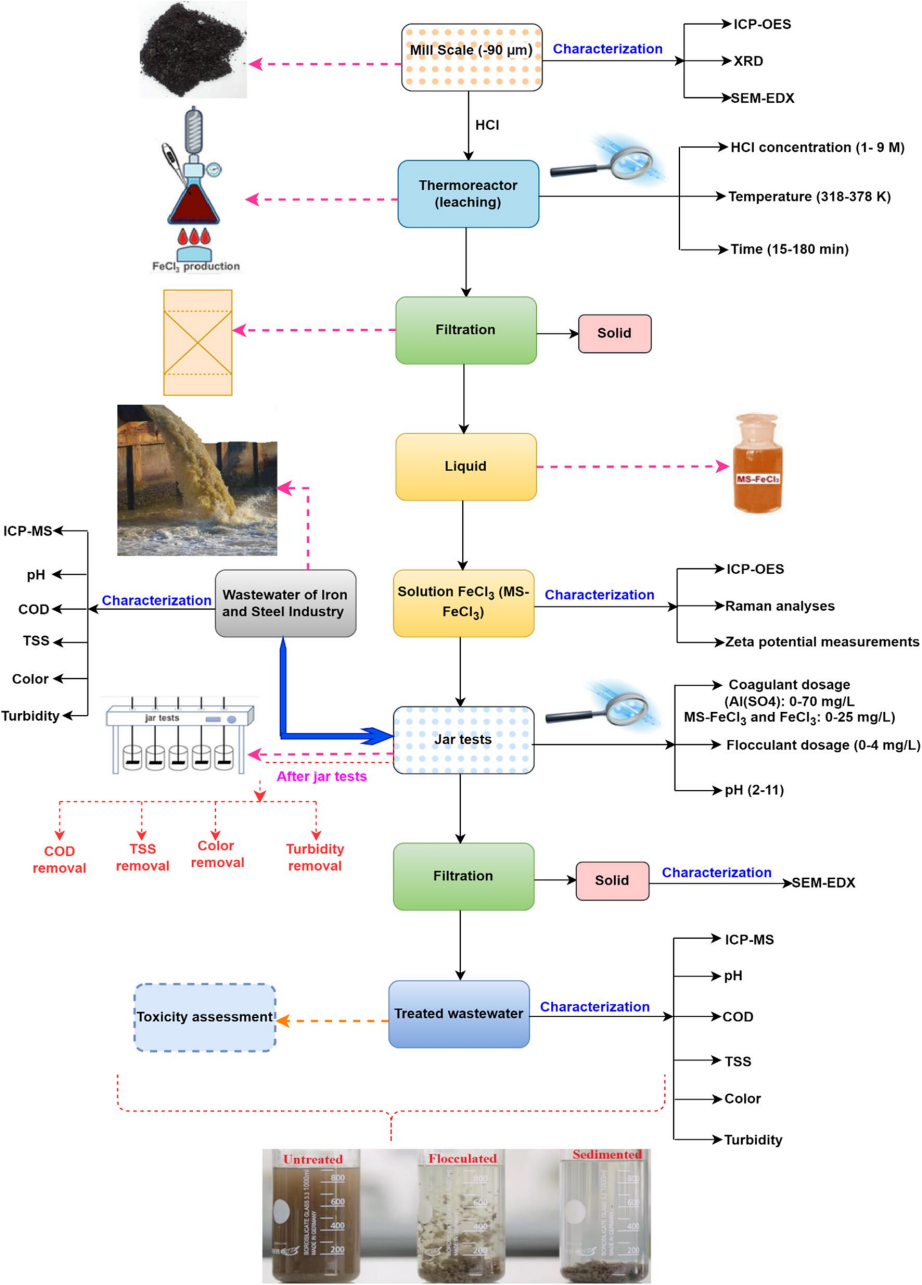
Proposed flow sheet mechanism for the treatment of I&SI using the coagulation method with FeCl_3_ produced from MS

The SEM images that display the morphological characteristics of the sludge, the elemental composition of the sludge based on weight (%), and the EDX spectra of the sludge obtained at optimum operating conditions using Al_2_(SO_4_)_3_, C-FeCl_3_, and MS-FeCl_3_ as coagulants are presented in Fig. 9 (a–c). The SEM images of the samples showed lumpy particles with an average size of several micrometers for different coagulants. The SEM images indicated that the solids had different forms and sizes and connected they formed flocs. The EDX spectra of the sludge obtained by using Al_2_(SO_4_)_3_, C-FeCl_3_, and MS-FeCl_3_ as coagulants showed the presence of Zn, Ca, Mg, Na, and Si in the sludge. Since some steel production operations are carried out using zinc-coated (galvanized) scraps, the zinc ion density in the plant’s wastewater was also high. These zinc ions were successfully precipitated, as seen in the elemental composition of the sludges. Additionally, it is thought that the element Si in the sludges come from the ore and raw materials used for slag-making during the production of raw iron. The carbon detected in the sludge corresponded to the carbon planchet used for the SEM micrographs, and the gold found in the sludge was associated with the gold coating used for the SEM process. Carbon and gold were not included in the total weight percentage calculations. When these elements were added, the cumulative percentage by weight become 100%. The results of the EDX analysis suggested that this procedure successfully recovered a substantial part of the inorganic species from the I&SI wastewater. A flowsheet mechanism of the processes designed for this study is illustrated in Fig. 10.

#### Comparison of results to literature

Information about some studies in which the removal of pollutants from iron-steel industry wastewater was carried out using the coagulation-flocculation method is presented in Table 5. In a previous study, in the steel production factory, the cooling liquid of the hot cylinder was treated, where Al_2_(SO_4_)_3_, FeCl_3_, and FeSO_4_ were used as coagulants at dosages ranging from 1.0 to 3.0 mg/L. Anionic, nonionic, and cationic polyelectrolytes were used as flocculants at concentrations of 4.0 mg/L. These coagulants were found to be quite efficient, and it was seen that they removed more than 98% of the TSS. Additionally, while the turbidity unit of the wastewater was 20 NTU, the turbidity decreased below 5 NTU after the removal processes carried out at the optimum dosages of Al_2_(SO_4_)_3_, FeCl_3_, and FeSO_4_. The authors emphasized that the addition of flocculant increased the efficiency of the treatment process (Kim et al. 2010). Similarly, in a study where automobile coating wastewater was treated using Al_2_(SO_4_)_3_ and CaO with the same method, it was reported that the removal efficiency was 97.1% in the turbidity parameter and 10.5% in the COD parameter (Xiong et al. 2017). In another study, synthetic and actual electroplating industry wastewater, which included heavy metals, was treated for the removal of heavy metals using FeCl_3_ at the coagulant, and it was reported that the resulting product met the effluent standards in terms of heavy metals (Puasa et al. 2021). In this study, success was achieved in the removal of organic and inorganic pollutants by conducting coagulation and flocculation experiments with commercial Al_2_(SO_4_)_3_ and C-FeCl_3_ as well as MS-FeCl_3_ obtained from a waste product namely MS in actual I&SI wastewater. The polyelectrolyte dosages used in the jar tests had similar effects on COD, TSS, color, and turbidity removal in all three settings with the aforementioned coagulants. When MS-FeCl_3_ and C-FeCl_3_ were used at the same dosages (1.29 mg/L), the COD removal efficiency result was around 60% at the same polyelectrolyte dosage (0.25 mg/L), whereas other parameters were similar at removal efficiency values around 85–90%.

**Table 5:** Comparison of experimental studies with the literature

	This study			(Xiong et al. 2017)	(Khan et al. 2014)	(Akbal and Camci 2010)	(Ríos et al. 1998)	(Zhang et al. 2022a)	(Kim et al. 2010)	(Puasa et al. 2021)		(Abujazar et al. 2022b)	(Abou-Elela et al. 2021)
Wastewater type	Iron-steel industry			Automobile coating wastewater	Metal etching industry	Metal plating	Steel and metal-finishing industries	Mo metallurgy industry	Steel manufacture	Electroplating industry (Synthetic and real)		Iron-steel factory	Metal finishing and electro-coating industry
Coagulant type	Al_2_(SO_4_)_3_	C-FeCl_3_	MS-FeCl_3_	Al_2_(SO_4_)_3_, CaO	FeCl_3_	Al_2_(SO_4_)_3_, FeCl_3_	CaCl_2_, AlCl_3_	FeCl_3_ with Humic acid	Al_2_(SO_4_)_3_, FeCl_3_, FeSO_4_	FeCl_3_	Rosehip seeds powder		FeCl_3_
Coagulant dosage range, mg/L	0–16	0–22	0–22	Al_2_(SO_4_)_3_: 0.5–3.0 CaO:0–1.0	2.1–15.5	0–2,000	CaCl_2_: 5000 AlCl_3_: 40,000	0–12	1.0–3.0	0.07–3.43 mL	1,000–10,000		variable
Optimum coagulant dosage, mg/L	4.05	1.29	1.29	Al_2_(SO_4_)_3_: 2.0 CaO:0.2	15.5	500	CaCl_2_: 10,000 AlCl_3_: 40,000	0.5	2.,0	-	1000		2483
Flocculant type	Anionic polyelectrolyte			-	-	-	-	-	Anionic, nonionic, cationic	Polyacrylamide		-	Anionic
Flocculant dosage range, mg/L	0–4.00			-	-	-	-	-	4,0	-		-	Not effective
Optimum flocculant dosage, mg/L	2.0	0.25	0.25	-	-	-	-	-	4,0	40	-		-
Initial pH	3–11			8.8	6.2	4–12	-	3–9	-	8–12		5–10	Unstable
Optimum pH	7.0	5.0	5.0	8.8	6.2	10–11	-	5	-	12	8.0		4.5–8.5
Working volume, mL	50			200	500	200	-	-	-	-		500	30 m^3^
pH adjusts	H_2_SO_4_ or NaOH			-	-	HCl or NaOH	-	-	-	H_2_SO_4_ or NaOH		H_2_SO_4_ or NaOH	-
Conclusion	High COD, color, TSS, and turbidity removal Also, high heavy metal removal			97.1% turbidity and 10.5%COD removal	34% TOC removal	99% Cu, Cr, and Ni removal	> 88% turbidity removal	99.6% Mo removal	30% turbidity removal	99% Cu, Zn, and Cd removal		> %73.7 COD, TSS, NH_3_–N, Mn, Fe, Zn, Al, and N removal	87% COD, 94%TSS and 92% oil and grease removal

### Scale-up potential and future perspectives for FeCl_3_ production and wastewater treatment

The coagulation-flocculation method is the most common treatment method used in the treatment of large-scale industrial wastewater. Its high efficiency, ease of operation, and low maintenance cost make this method popular today compared to other systems (Teh et al. 2016). It is convenient for treating industrial wastewaters originating from fields such as the textile, mining, metallurgy, chemistry, and food industries (Zhao et al. 2021; Tran et al. 2023). Coagulants such as Al_2_(SO_4_)_3_, FeCl_3_, Fe_2_(SO_4_)_3_, PAC, and PAFC are used in the implementation of the process (Verma et al. 2012). The production of these chemicals requires highly complex processes, and it is known to have high costs. While market prices vary day by day, the price of FeCl_3_ is approximately 0.30 US$/kg, and the price of Al_2_(SO_4_)_3_ is around 0.20 US$ /kg. Considering the amount of Al_2_(SO_4_)_3_ and FeCl_3_ consumed per unit m^3^ of our wastewater, figures are around 1.013 US$ for Al_2_(SO_4_)_3_ and 0.387 US$ for FeCl_3_ (excluding taxes and other technical equipment). On the other hand, MS, which originates and emerges from currently used iron-steel production processes and is an intermediate product, can be used as an alternative, thanks to the process we propose. This is because this way, a product which is considered waste would be returned to the economy. Additionally, a more straightforward and more accessible production method was developed in this study rather than a method involving complex processes. The stoichiometric proof of FeCl_3_ production from sludge was provided, and the feasibility of this process was demonstrated by analyses on the contents of metals, as well as elemental pollutants by conducting treatment studies of the wastewater of an actual facility. There was no difference between the operation of the rapid mixing ponds during the functioning of the wastewater treatment plant compared to the commercial product. Considering the market share of FeCl_3_ in the world, it is evident that the proposed process in the treatment of wastewater with high flow rates will be beneficial both in terms of carbon footprint and economically. On the other hand, the use of coagulants that pass into the sludge as a result of the treatment process should also be evaluated. The amount of acid consumed during recovery also affects the total cost. Additionally, alternative treatment methods for the neutralization and removal of other pollutants are very important due to the acidic character of the wastewater generated during recycling and the fact that these wastewaters may contain heavy metals or organic/inorganic pollutants (Xu et al. 2009; Chakraborty et al. 2020).

## Conclusion

This study investigated the treatment of MS, which has a high iron content and is a significant waste of the iron-steel industry, in the presence of HCl and usage conditions of the obtained solution in the treatment of iron and steel wastewater. The results are summarized as follows:

- In the experiments where MS was treated with HCl and under the optimum conditions (7 M HCl, leaching temperature 378 K, leaching time 120 min, solid–liquid ratio 1/10 (g/mL), particle size − 90 μm), 100% Fe solubility, approximately 77% Fe^3+^ conversion rate, and 52.53 g/L Fe^3+^ concentration were achieved.
- Turbidity, color, and TSS removal efficiency values were found to be above 80%, while COD removal efficiency was 68.52% at the dosage of 4.05 mg/L Al_2_(SO_4_)_3_ and 2.0 mg/L anionic polyelectrolyte.
- Both MS-FeCl_3_ and C-FeCl_3_ were applied at a dosage of 1.29 mg/L. The turbidity, color, and TSS removal efficiency values were above 70% at the 0.25 mg/L anionic polyelectrolyte dosage. The COD removal efficiency value for MS-FeCl_3_ was 66.59%, while this was 65.66% with C-FeCl_3_.
- Heavy metal removal efficiency values over 96% were obtained in the Al_2_(SO_4_)_3_ study in terms of Fe, Cr, Mn, Ni, Zn, Cd, Hg, and Pb, which were also examined under the optimum conditions. In the MS-FeCl_3_ and C- FeCl_3_ studies, a removal efficiency of over 94.50% was achieved.

Consequently, the results of this study showed that the treatment performance of MS-FeCl_3_ obtained from MS was higher than that of Al_2_(SO_4_)_3_ and similar to that of C-FeCl_3_. The results of the heavy metal analyses also showed that with MS-FeCl_3_ obtained from MS, one of the wastes of the iron and steel industry, the wastewater of this sector was treated, contributing to sustainable waste management. Furthermore, this method is considered to be important in terms of economic and environmental awareness to dispose of a waste, obtain a coagulant from this waste, and use this coagulant successfully in a of wastewater treatment process.

## Data Availability

The datasets generated during and/or analyzed during the current study are available from the corresponding author upon reasonable request.

## Declarations

All authors have read, understood, and have complied as applicable with the statement on “Ethical responsibilities of Authors” as found in the Instructions for Authors and are aware that with minor exceptions, no changes can be made to authorship once the paper is submitted.
